# Food price volatility and socio-economic inequalities in poor food consumption status during coronavirus disease-2019 lockdown among slum and non-slum households in urban Nansana municipality, Uganda

**DOI:** 10.1186/s12937-023-00836-x

**Published:** 2023-01-11

**Authors:** Edward Buzigi, Stephen Onakuse

**Affiliations:** 1 Department of Public Health & Nutrition, Faculty of Health Sciences, Victoria University, Kampala, Uganda; 2 Department of Nutritional Sciences & Dietetics, Kyambogo University, Kampala, Uganda; 3 Department of Food Business & Development, University College Cork, Cork, Republic of Ireland

**Keywords:** COVID-19 lockdown, Food consumption scores, Food consumption status, Staple food prices, Socio-economic inequalities, Slums, Non-slums

## Abstract

**Background:**

This study assessed staple food price volatility, household food consumption scores (FCS), poor household food consumption status and its association with socio-economic inequalities during enforcing and partial lifting of coronavirus disease-2019 (COVID-19) lockdown restrictions in slum and non-slum households (HHs) of Nansana municipality, Uganda.

**Methods:**

Repeated cross-sectional surveys were conducted during enforcing and partial lifting of COVID-19 lockdown restrictions. A total of 205 slum and 200 non-slum HHs were selected for the study. Telephone based interviews with HH heads were used to collect data on socio-economic factors. Data for FCS was collected using the World Food Programme FCS method. Prices for staple foods were collected by face-to-face interviews with food vendors from the local market. Mean staple food price differences before COVID-19 lockdown, during enforcing, and partial lifting of lockdown was tested by Analysis of variance with repeated measures. Multivariate logistic regression analysis was used to assess the association between socio-economic variables and poor food consumption status. A statistical test was considered significant at *p* < 0.05.

**Results:**

Mean staple food prices were significantly higher during enforcing COVID-19 total lockdown restrictions compared to either 1 week before lockdown or partial lifting of lockdown (*p* < 0.05). Mean FCS for staple cereals and legumes were significantly higher in slum HHs during COVID-19 lockdown compared to when the lockdown was partially lifted (*p* < 0.05). In slum HHs, the prevalence of poor food consumption status was significantly higher during partial lifting (55.1%) compared to total lockdown of COVID-19 (15.1%), *p* < 0.05. Among slum HHs during lockdown restrictions, food aid distribution was negatively associated with poor food consumption status (AOR: 0.4, 95% CI: 0.1–0.6), whilst being a daily wage earner was positively associated with poor food consumption status (AOR: 0.5, 95% CI: 0.1–0.6). During partial lifting of COVID-19 lockdown in slum HHs, poor food consumption status was positively associated with female headed HHs (AOR: 1.2, 95%CI: 1.1–1.6), daily wage earners (AOR: 3.2, 95% CI: 2.6–3.8), unemployment (AOR: 1.9, 95% CI: 1.5–2.1) and tenants (AOR: 2.4, 95% CI: 1.8–3.5). Female headed HHs, daily wage earners and tenants were positively associated with poor food consumption status either during enforcing or partial lifting of COVID-19 lockdown restrictions in non-slum HHs.

**Conclusion:**

Staple food prices increased during enforcing either the COVID-19 lockdown or partial lifting of the lockdown compared to before the lockdown. During the lockdown, food consumption improved in slum HHs that received food aid compared to those slum HHs that did not receive it. Household heads who were females, daily wage earners, unemployed, and tenants were at risk of poor food consumption status either in slum or non-slum, and therefore needed some form of food assistance either during enforcing or partial lifting of the lockdown.

**Supplementary Information:**

The online version contains supplementary material available at 10.1186/s12937-023-00836-x.

## Background

Coronavirus disease-2019 (COVID-19) is a novel respiratory tract infection caused by severe acute respiratory syndrome coronavirus 2 [1]. The COVID-19 was first reported in December 2019, in Wuhan, China. However, the disease has spread globally [2, 3]. The disease can spread from person to person through small droplets from the nose or mouth which are spread when a person with COVID-19 coughs or breathes out [1]. The COVID-19 droplets are either inhaled by uninfected persons or land on objects and surfaces around the person [4, 5]. People contract the disease when they inhale the droplets, whilst others contract COVID-19 by touching these objects or surfaces, then touching their eyes, nose or mouth [1, 4, 5]. Social distancing defined as avoiding large crowds, crowded public places, and maintaining at least 2 m of distance between one person and others, has been recognised as a feasible strategy to halt the spread of COVID-19 [1, 6, 7].

A national lockdown strategy characterised by restricting people’s movements by forcing them to stay at their homes has been found to be one of the most effective social distancing strategies to halt the spread of COVID-19 [7–9]. The COVID-19 lockdowns may be partial or total varying from country to country [9]. A total lockdown is characterised by nationwide travel blockade and quarantine policy that requires all public spaces, businesses, and transport to close [8]. The Ugandan government announced enforcing the COVID-19 total lockdown on 30th March 2020 [10, 11]. However, in early June 2020, the COVID-19 lockdown was partially lifted [11]. The COVID-19 total lockdown in Uganda was characterised by closing all daily income generating public business including restaurants, barber shops, schools, bars, tourism, hotels and public transport such as air transport, buses, taxis and boda-boda (motor bike) taxis [12]. Partial lifting of COVID-19 lockdown was characterised by limited opening of road transport such as buses and taxis. However, all other businesses remained closed except essential business such as food markets and health facilities that remained open during enforcing and partial lifting of the COVID-19 lockdown in Uganda [10].

Food security is defined as a situation when all people, at all times, have physical and economic access to sufficient, safe and nutritious food that meets their dietary needs and food preferences for an active and healthy life [13]. It is worth noting that the global recession, caused by COVID-19 lockdowns and other restrictions on business activity to control COVID-19 may affect food security by disrupting the food supply chain and subsequently increase food prices or lower people’s income and therefore, making them unable to purchase adequate food to achieve sustainable household food consumption [14–16]. However, the highest burden is expected to be on the urban poor HHs in low income countries of many African countries that survive on purchased food that reaches them through informal food supply [17, 18]. Furthermore, staple food price volatilities and several other socio-economic inequalities are key determinants of HH food consumption status and food insecurity during enforcing restrictions that aim to prevent the spread of disease epidemics in low and middle income countries of Asia and Africa [15, 16, 19, 20]. Informal settlements, commonly known as slums, are increasing in many urban areas of African countries including Uganda [21, 22]. Compared to non-slum HHs, slums in Uganda are characterised by heavily populated urban informal settlements where the HHs are characterized by substandard housing, unemployment, and low standard of living such as inadequate sanitation facilities [23, 24]. Slum HHs, already disproportionately affected by chronic poverty and food insecurity [25], are highly vulnerable to different forms of shocks, including those arising from enforcing COVID-19 lockdown restrictions [26]. However, there is scarce evidence on the impact of COVID-19 lockdown restrictions on food prices and household food consumption status in urban Uganda. Therefore, this study investigated the staple food price volatility, and the socio-economic inequalities of poor food consumption during enforcing and partial lifting of COVID-19 lockdown restrictions in slum and non-slum HHs of Nansana municipality, Wakiso district, Uganda.

## Methods

### Study setting and design

This study was conducted in Nansana Municipality located in urban Wakiso district, central Uganda, East Africa. Between June and August 2020, when the COVID-19 lockdown restrictions were enforced, 23 to 40% of the Uganda’s population was food insecure [27]. Moreover, 23% of the food insecure HHs were employing crisis coping strategies due to increasing food consumption gaps and reduced dietary diversity [27]. Nansana is among the fast-growing urban municipalities in Wakiso district harbouring both people of low and middle income status [28]. The total population of Nansana municipality is 365,124, of which 53 and 47% are females and males, respectively [28]. Furthermore, 17.3 and 2% of the population are children (below 5 years old) and elderly (65 years old) respectively. The total number of HHs are 90,742, of which 27% are female headed HHs [28]. The majority of urban dwellers in Uganda including Nansana Municipality are daily wage earners [28], residing in slum HHs characterized by informal and unplanned settlements with poor infrastructure and inadequate sanitation facilities [23, 24]. A repeated community cross-sectional study that was conducted twice in the same HH heads and local food vendors to ascertain data on HH food consumption scores/status and food prices, respectively. The first phase for collecting data on HH food consumption and staple food prices was between 14th and 27th April 2020, during the period when the COVID-19 lockdown was enforced. The second phase of data collection was done during the partial lifting of the COVID-19 lockdown, from 16th to 29th June 2020.

### Sample selection of participants from food markets and households

Food vendors were needed to provide prices for staple foods, maize and common bean. Food vendors were selected from Masitowa market, the central market that serves both slum and non-slum dwellers in Nansana municipality. The food vendors selling common bean and maize flour were purposively selected from the central market, Masitowa. In addition, HH heads were needed to provide information on HH food consumption.

A sample size of 401 households was estimated using the formula for single population proportion with the following assumptions: since we are doing the first ever prevalence study for poor food consumption status during the COVID-19 lockdown in Uganda, in such a situation, a 50% prevalence, P of the study condition is recommended for use [29], at 95% confidence level, with a 5% margin of error, and 5% allowance for possible non-response.

By using the formula n = N*X / (X + N – 1) where,

n is the minimum sample size of HHs needed for this study.

N = Number of households in the study area = 90,742 [28].

$$\textrm{X}=\frac{{\textrm{Z}}^2\ \textrm{P}\ \textrm{Q}}{{\textrm{D}}^2}$$

Z = Standard deviation score at 95% = 1.96.

P = Prevalence for a particular condition in a given population, when there is no previous studies to help estimate *P* = 50% = 0.5 [29].

Q = Complimentary probability (1-P) = 1–0.5 = 0.5.

D = Error margin = 5% = 0.05, substituting $\textrm{X}=\frac{1.96^2\textrm{x}0.5\textrm{x}0.5=384}{0.05^{2.}}$

Therefore, *n* = 90742*384/ (384 + 90,742–1) =382. However, if a 5% allowance for possible non-response is considered, then a total sample of 402 households was needed. Then, at least 201 HHs from either slum or non-slum place of residence were needed for recruitment in this study.

All vendors in the study local markets selling staple maize flour and common bean were purposively selected for the study. Food items, maize and common bean were selected for assessing food price volatility because they are the main staples in the study area. Moreover, the Ugandan government distributed maize flour and common bean to slum households as food aid. To select study participants who provided data on food consumption score (FCS), a multistage sampling procedure was used [30]. At the first stage, all local councils in Nansana municipality were listed and divided into categories as slum and non-slum local councils. Compared to a non- slum council, a slum council was defined as a heavily populated urban informal settlement where the inhabitants are characterized by substandard housing and low standard of living [23, 24]. From these lists, one slum local council, Kattoke was purposively selected because it received food aid items during enforcing the COVID-19 lockdown restrictions. Furthermore, one non-slum local council, Nansana West 1 was purposively selected because it did not receive food aid; it shares boundaries and a central market with the selected slum local council. At the last stage, HH heads from slum and non-slum local councils were recruited by snowball sampling. Snowball sampling is where research participants provide contacts for other eligible participants for the study [31]. Snowball sampling was deemed appropriate because most HHs in these areas have no formal addresses and phone directories to create a list for random sampling. Two research assistants experienced in telephone surveys were assigned to each of the two study local councils. After interviewing each HH head, the mobile phone contacts of the next participant in the neighbourhood HHs were obtained by snowball sampling. On giving a telephone call, and before participating in the study, potential participants were first asked the council they reside. The participant was not recruited if he/she did not belong to the eligible council. In situations when the participant did not reside in the eligible council, the previous participant was contacted to avail the telephone contacts of another potential participant. All participants were available to be reached by phone. However, some study participants were not available in the time they were scheduled for the telephone interviews. Therefore, based on the time frame of the study, respondents were given an opportunity to schedule a convenient time for their availability to participate in the telephone interview. The convenient time selected by such respondents was adhered to by the interviewers. Telephone interviews were preferred to face–face interview because it was necessary to follow the COVID-19 prevention guidelines of ensuring social distancing [32, 33].

### Data collection and measurement of study variables

#### Outcome variables

The outcome variables of this study were staple food prices, food consumption scores, and poor food consumption status.

#### Measurement of staple food prices

Food markets were entitled to remain open during the period of enforcing and partial lifting of the COVID-19 lockdown in Uganda. Therefore, by observing the Uganda’s Ministry of Health guidelines of social distancing and wearing of face masks to halt the spread of COVID-19, trained research assistants visited food sellers in their food markets during the periods of enforcing and partial lifting of COVID-19 lockdown to enquire about the price of common bean (dry beans) and maize flour. Food price data were collected on common bean and maize flour because they are major food staples in Uganda [34, 35]. Moreover, the Ugandan government decided to distribute staple common beans and maize flour as food aid to the vulnerable poor in urban districts of Kampala and Wakiso during the COVID-19 lockdown [36].

Staple food prices were enquired for the periods of 1 week before COVID-19 lockdown, during enforcing COVID-19 lockdown restrictions, and during partial lifting of lockdown. The first visit of the food markets was 14th to 15th April 2020, during the period when the COVID-19 lockdown was enforced. The food prices during COVID-19 lockdown and a week before COVID-19 lockdown were enquired during the first visit. The questions asked during the first market visit were “how much do you sell a kilogram of common bean; and maize flour now?” “What was the price of a kilogram of common bean and maize flour one week before the COVID-19 lockdown was enforced by government?” Furthermore, the staple food prices during the partial lifting of the lockdown were enquired during the second visit (16th to 17th June 2020). The question asked was “how much do you sell a kilogram of common bean; and maize flour now?” It is worth noting that both visits were done in the same central food market as explained by the World Food Programme (WFP) market analysis tool [37].

#### Measurement of food consumption scores and food consumption status

Food consumption scores and poor food consumption status were measured by the World Food Programme FCS method [38]. The FCS method uses a brief questionnaire to ask respondents about the frequency of their household’s consumption of eight different food groups over the preceding 7 days. To calculate the FCS from these results, the consumption frequencies are summed and multiplied by the standardized food group weight as shown in table 1.

**Table 1:** Food groups and their corresponding weights used in the food consumption score method

	Food group	Weight
1	Main staples, cereals and tubers	2
2	Pulses	3
3	Vegetables	1
4	Fruits	1
5	Meat/fish	4
6	Milk	4
7	Sugar	0.5
8	Oil	0.5

For calculating food consumption status, HHs were further classified as having “poor,” “borderline,” and “acceptable” food consumption status by applying the WFP recommended cut-offs to the FCS of 0–21, 21.5–35, and > 35, respectively [38]. The following steps were followed while measuring FCS and food consumption status for the study HHs as described by World Food Programme [38]. 1. Group food items in the specified food groups (condiments not included); 2. Sum all the consumption frequencies of food items within the same group; 3. Multiply the value of each food group by its weight (see table 1); 4. Sum the weighted food group scores to obtain FCS; 5. Determine the household’s food consumption status based on the following thresholds: 0–21: Poor; 21.5–35: Borderline; > 35: Acceptable. A binary outcome (yes/no) for poor food consumption status, FCS of 0–21 was created.

### Exposure variables

The exposure variables included period when there was either total COVID-19 lockdown restrictions or when the lockdown was partially lifted. Therefore, this study compared food prices, FCS, and HH food consumption status across the periods of enforcing and partial lifting of COVID-19 lockdown restrictions. Another exposure variable was residence in either slum or non-slum HHs of urban Nansana Municipality, Wakiso district. A comparison between slum and non-slum HHs was necessary because Uganda’s urban slum dwellers are disproportionately affected by poverty [25], and shocks such as epidemics that negatively impact on HH food consumption and food security [39].

### Covariates

Food consumption scores and food consumption status were assessed at the HH level. Therefore, all the covariates used in this study were captured and measured at the HH level as well. Socio-economic covariates such as sex of HH head, source of income for HH head, size of HH, age of HH heads, HH head ownership of house, and HH receiving food aid were considered for this study because they have been found to influence HH food consumption either during disease disasters or without disease disasters [39, 40]. Furthermore, HH receipt of food aid was considered as a covariate because the government of Uganda through the president had promised to give food aid to vulnerable poor HHs in Wakiso district, including slum HHs located in Nansana municipality during the COVID-19 lockdown [41].

### Statistical data analysis

Data analysis was done by STATA, version 15.0 at *p* < 0.05. Differences in mean staple food prices between the three time periods, 1 week before enforcing total COVID-9 lockdown; during enforcing lockdown; and partial lifting of lockdown was analysed by Analysis of variance (ANOVA) with repeated measures. Mean FCS differences in either slum or non-slum HHs between enforcing and partial lifting of COVID-19 lockdown restrictions was analysed by a paired t test. Mean food consumption status differences between slum and non-slum HHs were analysed by unpaired t test. A binary outcome of poor food consumption status, FCS of 0–21 and no poor food consumption status, FCS of ≥21.5 was created [38]. The association between socio-economic variables and poor food consumption status was tested using multivariate logistic regression analysis. An odds ratio (OR) of socio-economic variables and 95% confidence interval (CI) was computed by multivariate logistic regression analysis. Differences in prevalence of poor food consumption status between COVID-19 lockdown and partial lifting of lockdown was analysed using McNemar’s test.

### Ethical considerations

Permission to carry out the study was granted by the Office of the Mayor, Nansana Municipality. Ethical approval was granted by The AIDS Support Organisation Research Ethical Committee (Reference number TASO-REC/066/19-UG-REC-009). Prior to any enrolment to the study, informed and verbal consent was sought and obtained from study participants through phone calls. Signed consent was not obtained individually from study participants because of the COVID-19 lockdown restrictions such as social distancing and avoiding physical contact with study participants.

## Results

### Background characteristic of study participants

Data were collected twice among the same central market and HHs during enforcing and partial lifting of COVID-19 lockdown. It is worth noting that 128 food vendors were recruited in the first market visit survey. However, one food vendor was lost to follow-up in the second market visit survey. Therefore data for 127 food vendors who participated in both market visit surveys were included in the analysis. Out of the 127 food vendors, 65 (51.2%) and 62 (48.8%) were selling staple common bean and maize, respectively. Out of the 127 food sellers, 75 (59%) and 52 (41%) were females and males, respectively. In addition, a total of 208 and 202 slum and non-slum HH heads, respectively participated in data collection during COVID-19 lockdown. However, 3 of 208 and 2 of 202 slum and non- slum HHs respectively were lost to follow-up on the second phase of data collection (period when COVID-19 lockdown restrictions were partially eased). Therefore, data for 205 and 200 slum and non-slum HHs were available for analysis during the period of enforcing and partial lifting of the COVID-19 lockdown.

### Staple food prices before, during enforcing and partial lifting of COVID-19 lockdown

Maize flour and common bean are major staple food legumes and cereals in Uganda, including the study area. Table 2 shows the association between the period of COVID-19 lockdown restrictions and the price of staple foods, maize flour and common bean in Nansana municipality, Uganda.

**Table 2:** Prices for staple foods before, during enforcing and partial lifting of COVID-19 lockdown

Period of COVID-19 lockdown	Mean price/kg in Uganda shillings	
	Maize flour Mean ± SD	Common bean Mean ± SD
1 week before lockdown	2030 ± 25^a^	2558 ± 45^a^
Enforcing lockdown	2383 ± 30^b^	4333 ± 40^b^
Partial lifting of lockdown	1823 ± 26^a^	2847 ± 30^c^

Values in the same column with different superscript letters are significantly different (*p* < 0.05) according to the repeated measures Analysis of Variance with Bonferroni corrected test; Kg: Kilogram.

The mean price of maize flour was significantly higher, 2383 Uganda Shillings (Ug Shs) during enforcing COVID-19 lockdown restrictions compared to before lockdown, 2030 Ug Shs and partial lifting of COVID-19 lockdown restrictions, 1823 Ug Shs (*p* < 0.05). The price of common bean was significantly higher, 4333 Ug Shs during enforcing COVID-19 lockdown restrictions compared to before, 2558 Ug Shs and partial lifting, 2847 Ug Shs of the COVID-19 lockdown restrictions (*p* < 0.05). The price of common bean was significantly lower (2847 Ug Shs) during partial lifting of COVID-19 lockdown restrictions compared to when there was total lockdown (4333 Ug Shs). In contrast, the price of common bean was significantly higher, 2847 Ug Shs during partial lifting of COVID-19 lockdown compared to 1 week before enforcing the lockdown restrictions, 2558 Ug Shs (*p* < 0.05).

#### Household food group consumption scores during enforcing and partial lifting of COVID-19 lockdown restrictions

The WFP method for measuring FCS uses eight food groups including staple cereals/tubers, legumes/pulses, fish/meat, milk, fruits, vegetables, sugar and oil. Table 3. Shows the food group consumption scores among slum and non-slum households during enforcing and partial lifting of COVID-19 lockdown restrictions. The total FCS of non-slum HHs was at borderline (34.9) and acceptable (37.3) during lockdown and partial lifting of lockdown, respectively. In contrast, the total FCS of slum HHs was at borderline (21.5–35) either during lockdown or partial lifting of lockdown as shown in table 3 .

**Table 3:** Food group consumption scores among slum and non-slum households during enforcing and partial lifting of COVID-19 lockdown restrictions

Food group	Slum HHs, *N* = 205			Non Slum HHs, *N* = 200		
	Lockdown	Partial lockdown		Lockdown	Partial lockdown	
	FCS ± SD	FCS ± SD	P	FCS ± SD	FCS ± SD	P
Cereals/tubers	10.56 ± 0.02	8.91 ± 0.04	0.02*	10.61 ± 0.03	11.1 ± 0.04**	0.4
Pulses	14.65 ± 0.01	12.81 ± 0.02	0.03*	14.79 ± 0.04	15.1 ± 0.05**	0.3
Vegetables	0.50 ± 0.01	0.49 ± 0.04	0.1	0.93 ± 0.06**	1.03 ± 0.05**	0.7
Fruit	0.41 ± 0.02	0.42 ± 0.03	0.29	0.42 ± 0.03	0.44 ± 0.02	0.4
Meat-Fish	0.28 ± 0.04	0.29 ± 0.04	0.8	0.89 ± 0.02**	1.52 ± 0.03**	0.03*
Milk	0.41 ± 0.05	0.43 ± 0.04	0.8	1.21 ± 0.04**	1.98 ± 0.05**	0.02*
Sugar	1.95 ± 0.04	1.96 ± 0.05	0.9	2.99 ± 0.03**	3.10 ± 0.02**	0.4
Oil	1.96 ± 0.03	1.98 ± 0.03	0.7	3.02 ± 0.02**	3.07 ± 0.01**	0.2
Total FCS	**30.7**	**27.3**		**34.9**	**37.3**	

FCS: Food consumption scores are reported in means; SD: Standard Deviation; HH: Household; P: Probability value.*Food consumption scores are significantly different between enforcing and partial lifting of lockdown in the same households. **Food consumption scores are significantly different between slum and non-slum HHs either during enforcing or partial lifting of lockdown restrictions. Total FCS: 0–21 is poor; 21.5–35 is borderline; > 35 is acceptable

The mean FCS for staple cereals/tubers were significantly higher for slum HHs during enforcing COVID-19 restrictions (FCS, 10.6) compared to when the COVID-19 restrictions were partially lifted (FCS, 8.9), *p* < 0.05. Furthermore, mean FCS for pulses were significantly higher for slum HHs during enforcing COVID-19 restrictions (FCS, 14.7) compared to when the COVID-19 restrictions were partially lifted (FCS, 12.8) at *p* < 0.05. The mean FCS of the other six food groups was not significantly different from each other in slum HHs between enforcing and partial lifting of COVID-19 lockdown restrictions. For non-slum HHs, the mean FCS were significantly higher for meat-fish and milk during partial lifting of COVID-19 lockdown restrictions compared to the period when there was total lockdown restrictions (*p* < 0.05).

Furthermore, the mean FCS for vegetables, meat/fish, milk and sugar were significantly higher in non-slum HHs compared to slum HHs during enforcing of COVID-19 lockdown restrictions (p < 0.05). There was no significant difference in the FCS for cereals/tubers, pulses and fruits between slum and non-slum HHs during enforcing COVID-19 lockdown restrictions. On partial lifting of the COVID-19 lockdown, the FCS was significantly higher for cereals/tubers, pulses/legumes, milk, meat/fish, vegetables, oil and sugar in non-slum HHs compared to slum HHs (*p* < 0.005). However, the FCS for fruits was not significantly different between slum and non-slum HHs during partial lifting of the COVID-19 lockdown restrictions (*p* > 0.05).

### Characteristics associated with poor food consumption status during enforcing and partial lifting of COVID-19 restrictions among slum HHs

Table 4 shows the bivariate (unadjusted) and multivariate (adjusted) analysis of characteristics associated with poor food consumption status among slum HHs during enforcing and partial lifting of COVID-19 lockdown restrictions.

**Table 4:** Bivariate and multivariate logistic regression analysis of factors associated with poor food consumption status during enforcing and partial lifting of COVID-19 lockdown among 205 slum households in Nansana municipality, Wakiso district, Uganda

Variables (n)	Total lockdown				Partial lifting of lockdown			
	Poor food consumption status		COR (95%CI)	AOR (95% CI)	Poor food consumption status		COR (95% CI)	AOR (95% CI)
	Yes n (%)	No n (%)			Yes n (%)	No n (%)		
HH (205)	31(15.1)	174(84.9)			113(55.1)**	92(44.9)		
Sex of HH head								
Female (57)	7(12.3)	50 (87.7)	1.2(0.7–3.3)	1.2(0.6–3.3	37(69.4)	20(30.6)	1.4(1.2–1.8)*	1.2(1.1–1.6)*
Male (148)	24(11.7)	124(89.3)	1.00 (ref)	1.00 (ref)	76(51.4)	72(48.6)	1.00 (ref)	1.00 (ref)
Age range								
18–24 (68)	10(14.7)	58(85.3)	1.00 (ref)	1.00 (ref)	38(55.9)	30(44.1)	1.00 (ref)	1.00 (ref)
25–44 (104)	16(15.3)	88(84.7)	1.1(0.8–2.1)	1.2(0.7–2.2)	57(54.8)	47(45.2)	1.1(0.8–1.3)	1.1(0.7–1.3)
≥ 45(33)	5(15.1)	28 (84.9)	0.9(0.6–1.7)	0.8(0.7–1.8)	18(54.5)	15(45.5)	1.1(0.9–1.3)	1.1(0.8–1.2)
HH size								
1–5 (149)	23(15.4)	126(84.6)	1.00 (ref)	1.00 (ref)	73(49)	76(51)	1.00 (ref)	1.00 (ref)
6–10 (56)	8(14.3)	48(85.7)	1.2(0.7–1.3)	1.3(0.6–1.4)	40(71.4)	16(28.6)	1.3(1.1–1.5)*	1.2 (0.9–1.2)
Occupation^c^								
Daily wage^a^(99)	15(15.2)	84 (84.8)	1.2(1.1–5.2)*	1.1(0.8–4.3)	71 (71.7)	28(28.3)	3.4(2.8–4.1)*	3.2(2.6–3.8)*
Unemployed^b^ (56)	9(16.1)	47 (83.9)	1.1(0.8–7.9)	1.1(0.7–7.7)	30 (53.6)	26(46.4)	2.2(1.4–2.6)*	1.9(1.5–2.1)*
Formal Employment (50)	7(14.0)	43(84.0)	1.00 (ref)	1.00 (ref)	12(24)	38(76)	1.00 (ref)	1.00 (ref)
Education status								
None (36)	5(13.9)	31(86.1)	1.00 (ref)	1.00(ref)	20(55.6)	16(44.4)	1.00(ref)	1.00 (ref)
Primary (71)	11 (15.5)	60 (84.5)	1.1(0.7–1.3)	1.1(0.8–1.3)	40(54.9)	31(43.7)	1.2(0.8–1.1)	1.1(0.9–1.2)
Secondary (62)	9(14.5)	53(85.5)	0.9(0.8–1.3)	1.1(0.9–1.2)	33(53.2)	29(46.7)	1.1(0.9–1.2)	1.2(0.8–1.3)
Tertiary (36)	6(16.7)	30(83.3)	1.2(0.7–1.2)	1.2(0.8–1.2)	20(55.6)	16(44.4)	1.2(0.8–1.3)	1.1(0.9–1.2)
Received food aid								
Yes (179)	7(3.9)	172(96.1)	0.6(0.3–0.7)*	0.4(0.1–0.6)*				
No (26)	22(84.6)	4(15.4)	1.00 (ref)	1.00(ref)				
House ownership								
Owner (21)	4 (19)	17(81)	1.00 (ref)	1.00 (ref)	3(14.3)	18 (25)	1.00(ref)	
Tenant (184)	27(14.7)	157(85.3)	1.8(0.8–2.1)	1.7(0.8–2.2)	110(59.8)	74(48.2)	2.7(1.9–3.7)*	2.4(1.8–3.5)*

HH = Household, COR = Crude Odds Ratio, AOR = Adjusted Odds Ratio, CI = Confidence Interval; *Significant association at 95% CI and *p* < 0.05; ^a^ means someone in informal employment and the compensation he/she receives as wages for services performed during a business day; ^b^ Includes those who get remittance as source of income; ** a paired t test indicated that the prevalence of poor food consumption is significantly different between enforcing and partial lifting of the COVID-19 restrictions at *p* < 00.5; ^c^ occupation status on the day of entering lockdown.

After, conducting a McNemar’s test, the prevalence of poor food consumption status among slum HHs was significantly higher during partial lifting of COVID-19 lockdown restrictions (55%) compared to the period when the COVID-19 lockdown restrictions were totally enforced (15%) (*p* < 0.05). The unadjusted analysis during enforcing COVID-19 lockdown restrictions showed that daily wage earners were 1.2 times likely to have HHs with poor food consumption status compared to HH heads who had formal employment and unemployed (COR 1.2, 95% CI: 1.1–5.2, p < 0.05). However, in adjusted analysis, the association between daily wage earners and poor food consumption status was not significant (AOR 1.1, 95% CI: 0.8–4.3, *p* > 0.05). The slum HHs that received food aid were less likely to have poor food consumption status in both unadjusted (COR 0.6, 95% CI: 0.3–0.7, *p* < 0.05) and adjusted (AOR 0.4, 95% CI: 0.1–0.6, p < 0.05) analysis compared to HHs that did not receive food aid. All the other socio-economic characteristics were not significantly associated with poor food consumption status among slum HHs during enforcing COVID-19 lockdown restrictions. The prevalence of poor food consumption status increased from 15.1% during enforcing COVID-19 lockdown restriction to 55.1% during partial lifting of COVID-19 lockdown restrictions. Female headed HHs, daily wage earners, unemployed, and tenants were positively associated with poor food consumption at AOR 1.2 (CI: 1.1–1.6), AOR 3.2 (CI: 2.6–3.8), AOR 1.9 (1.5–2.1), and AOR 2.4 (CI: 1.8–3.5), respectively.

### Characteristics associated with poor food consumption status during enforcing and lifting COVID-19 restrictions among non-slum HHs

Table 5 shows the bivariate and multivariate logistic regression analysis of factors associated with food consumption status during enforcing and partial lifting of COVID-19 lockdown among 200 non-slum households in Nansana municipality, Wakiso district, Uganda.

**Table 5:** Bivariate and multivariate logistic regression analysis of factors associated with poor food consumption status during enforcing and partial lifting of COVID-19 lockdown among 200 non- slum households in Nansana municipality, Wakiso district, Uganda

Variables (n)	Lockdown				Partial lifting of lockdown			
	Poor food consumption status		COR (95%CI)	AOR (95% CI)	Poor food consumption status		COR (95%CI)	AOR (95%CI)
	Yes n (%)	No n (%)			Yes n(%)	No n(%)		
HH (200)	29(14.5)	171(85.5)			25(12.5)	175(87.5)		
Sex of HH head								
Female (49)	9(18.4)	40 (81.6)	1.3(1.1–2.5)*	1.2(1.1–2.3)*	6 (12.2)	43(87.8)	1.1(0.9–1.3)	1.1(0.8–1.3)
Male (151)	20(13.2)	131(86.8)	1.00 (ref)	1.00 (ref)	19(12.6)	129(85.4)	1.00 (ref)	1.00 (ref)
Age range								
18–24 (16)	2(12.5)	14(87.5)	1.00 (ref)	1.00 (ref)	2(12.5)	14(87.5)	1.00 (ref)	1.00 (ref)
25–44 (154)	23(14.9)	131(85.1)	1.2(0.8–1.4)	1.2(0.9–1.3)	20(13.0)	134 (83)	1.1(0.8–1.3)	1.1(0.9–1.4)
≥ 45 (30)	4(13.3)	26(82.7)	1.1(0.9–1.2)	1.1(0.8–1.2)	3(13.3)	27(82.7)	1.1(0.7–1.2)	1.1(0.6–1.3)
HH size								
1–5 (157)	23(14.6)	134(85.4)	1.1(0.9–1.2)	1.2(1.1–1.3)	21(13.4)	136(86.7)	0.8(0.7–0.9)*	1.1(0.8–1.1)
6–10 (43)	6(14)	37(86.0)	1.00 (ref)	1.00 (ref)	4(9.3)	39(82.7)	1.00 (ref)	1.00 (ref)
Occupation^c^								
Daily wage^a^ (70)	17(24.3)	53(75.7)	1.3(1.2–1.9)*	1.2(1.1–1.5)*	15(21.4)	55 (78.6)	1.3(1.1–1.8)*	1.2(1.1–1.6)*
Unemployed^b^(24)	10(41.7)	14(58.3)	1.4(1.2–4.3)*	1.3(1.1–3.8)*	9(37.5)	15(62.5)	1.5(1.3–2.2)*	1.4(1.2–1.9)*
Formal Employment (106)	2(1.9)	104(98.1)	1.00 (ref)	1.00 (ref)	1(0.94)	105(99.6)	1.00 (ref)	1.00 (ref)
Education status								
None (11)	2(18.2)	9(81.8)	1.00 (ref)	1.00 (ref)	3(27.3)	8(72.7)	1.00 (ref)	1.00 (ref)
Primary (23)	4(17.4)	19(82.6)	1.2(0.6–1.2)	1.1(0.7–1.2)	6(26.1)	17(73.9)	1.6(0.8–1.5)	1.5(0.7–1.4)
Secondary (39)	7(17.9)	32(82.1)	1.2(0.4–1.3)	1.1(0.3–1.3)	10(25.6)	29(74.4)	1.6(0.6–1.5)	1.5(0.5–1.4)
Tertiary(127)	16(12.6)	111(87.4)	0.4(0.3–0.8)*	0.2(0.1–0.6)*	6(4.7)	121(95.3)	2.1(2.3–2.9)*	1.8(1.6–2.6)*
House ownership								
Owner (102)	10(9.8)	92(90.2)	1.00(ref)	1.00 (ref)	8(7.8)	94(92.2)	1.00 (ref)	1.00 (ref)
Tenant (92)	19(20.7)	69(79.3)	1.4(1.3–2.4)	1.3(1.1–2.1)	17(18.5)	81(81.5)	1.3(1.2–2.4)*	1.2(1.1–2.1)*

HH = Household, COR = Crude Odds Ratio, AOR = Adjusted Odds Ratio, CI = Confidence Interval; *Significant association at 95% CI and *p* < 0.05; ^a^ means someone in informal employment and the compensation he/she receives as wages for services performed during a business day; ^b^ Includes those who get remittance as a source of income; ^c^occupation status on the day of entering lockdown.

The prevalence of poor food consumption among non-slum HHs reduced from 14.5% during enforcing COVID-19 lockdown restrictions to 12.5%, when the lockdown restrictions were partially eased. However, the difference in the prevalence of poor food consumption between during enforcing total lockdown and partial lockdown was not significantly different from each other. Female headed HHs, daily wage earners, unemployed, and tenants were most likely to have poor food consumption at AOR 1.3 (CI: 1.1–2.5), AOR 1.2 (CI: 1.1–1.5), and AOR 1.3 (CI: 1.1–1.5), and AOR 1.3(1.1–2.1), respectively during enforcing COVID-19 lockdown restrictions. On partial lifting of the COVID-19 lockdown restrictions, daily wage earners, unemployed and tenants remained associated with poor food consumption at AOR 1.2(CI: 1.1–1.6), AOR 1.4(CI: 1.2–1.9), and AOR1.2(CI:1.1–2.1), respectively.

## Discussion

### Price volatility of staple foods

This study demonstrates that the price of staple common bean and maize flour was significantly higher during enforcing the COVID-19 lockdown restrictions compared to just before the lock down, and when the lockdown restrictions were partially eased. Similar findings of increased food prices during enforcing COVID-19 lockdown restrictions were reported in China [42] and some European countries [43]. The significantly higher staple food prices observed during enforcing the COVID-19 restriction in this present study could be attributed to the interrupted food supply chain due limited transportation of food from rural to urban centres of Nansana municipality, leading to low supply of food in the market amidst a high demand as reported elsewhere [44]. It is worth noting that food merchants in Uganda use cheap informal transport systems such as public transport vehicles including taxis and buses to transport both food and passengers from rural to urban areas [17]. However, public transport was suspended during the COVID-19 lockdown in Uganda [45], which might have contributed to the interrupted food supply chain from rural to urban centres such as Nansana municipality. Moreover, private vehicles such as trucks might have been more expensive to individual small-scale farmers and food merchants to contract and transport food from rural to urban centres.

### Food group consumption scores and food consumption status correlates for slum and non-slum households during enforcing and partial lifting of COVID-19 lockdown

Food consumption scores for cereals/tubers and pulses/legumes were significantly higher among slum HHs during enforcing COVID-19 lockdown restrictions compared to when the lockdown was partially eased. This could be attributed to the food aid items, maize flour (cereal) and common bean (legume) distributed to the slum HHs during when the COVID-19 restrictions were enforced [36, 46]. The prevalence of poor food consumption status was significantly lower (15%) during enforcing COVID-19 lockdown compared to partial easing of the lockdown (55%), probably because there was food aid distribution in the former and no food distribution in the latter. It is worth noting that food aid distribution was targeted to slum HHs in urban areas of Kampala and Wakiso districts including Nansana Municipality [47]. However, this study revealed that 13% (26/205) of the slum HHs did not receive food aid items. This could explain the 15% prevalence of poor food consumption status observed in slum HHs during enforcing COVID-19 lockdown measures.

Some slum HHs did not receive food aid items because they were insufficient to serve all the slum HHs, and this could be attributed to the bribery and embezzlement of funds intended to procure food items for distributing to slum HHs [47]. It was established that officials from the office of the prime minister might have inflated food prices and rejected lower price offers from various suppliers of maize flour and common beans [47], a situation which might have led to procurement and supply of low amounts and poor quality of food aid. To prevent such corruption and embezzlement of funds associated with food aid items procurement and distribution in the face of limited access to food during COVID-19 lockdown, cash transfers have been suggested as a better strategy compared to food aid distribution [48–50]. This is plausible because cash transfers are less prone to corruption since funds pass through fewer middlemen, thus limiting the number of officials with discretionary powers and private interests [49]. It is worth noting that mobile telephone money cash transfers would be feasible because over 70% of Ugandans own mobile phones [51]. Other countries such as South Africa [52], Kenya [53], and India [54] have successfully used cash transfer as a form of food assistance programme during the COVID-19 lockdown restrictions. Besides, food markets remained open and functional during the COVID-19 lockdown in Uganda. Therefore, the money received through cash transfer could be used by HH members to procure a variety of foods from different food groups from the local market to improve on their HH dietary diversity.

The mean FCS for vegetables, meat/fish, milk and sugar were significantly higher in non-slum HHs compared to slum HHs during either enforcing or partial lifting of COVID-19 lockdown restrictions. This may explain the lower prevalence of poor food consumption status observed in non-slum HHs compared to the slum HHs. This is plausible because FCS is a composite score based on dietary diversity, food frequency, and the relative nutritional importance (weight) of different food groups. Therefore, food groups significantly consumed by non-slum HHs such as milk and meat/fish are given a maximum weight of 4 each compared to the weight of 2 and 3 for staple cereals (maize) and legumes (common bean), respectively that were given as food aid and predominantly consumed by slum HHs.

As expected, slum HHs that received food aid items were less likely to have poor food consumption status. There was no significant association between poor food consumption status and socio-economic characteristics among slum HHs during enforcing of COVID-19 lockdown restrictions probably because slum HHs received some form of food aid items during this period.

In contrast, poor food consumption status during partial lifting of COVID-19 restrictions was significantly associated with HHs headed by women, daily wage earners, unemployed and tenants from slum HHs. The high prevalence of poor food consumption status observed in slum HHs may suggest that there was need to continue with food aid distribution during partial lifting of COVID-19 lockdown, with emphasis on HHs at risk such as female headed HHs, daily wage earners, unemployed and tenants. In addition, the positive association between poor food consumption and female headed HHs, daily wage earners, unemployed and tenants among non-slum HHs either during enforcing or partial lifting of COVID-19 lockdown restrictions could echo that some HHs in slums such as those whose heads were females, daily wage earners, unemployed and tenants needed some form of food assistance during the periods of lockdown.

The positive association between female headed HHs and poor food consumption status could be explained by the fact that the vast majority of women in Uganda are employed in the informal daily wage earning sectors and therefore, it is likely that the COVID-19 quarantine restrictions significantly reduced women’s economic and livelihood activities, increasing loss of incomes, and inadequate access to food [10, 55]. The poor food consumption status established in female headed HHs in this study is consistent with other studies from Afghanistan [56] and Uganda [10], which showed that female HH heads raised a concern that hunger would kill their family members before COVID − 19 does. Findings from this present study demonstrate gender inequality in HH food consumption during COVID-19 lockdown restrictions. The positive association between daily wage earners and poor food consumption status is plausible because businesses for daily wage earners such as motorbike tax riders, minibus taxi riders/conductors, barbers; arcade shop attendants, bar and hotel or guest house attendants remained closed during partial lifting of the COVID-19 lockdown in Uganda [45, 57]. Therefore, there was reduced access to incomes among urban daily wage earners in Nansana municipality, making it difficult for them to procure food items for their HH members [45, 57]. The unemployed HH heads were likely to have poor HH food consumption status because they lacked any form of income generating activity during either the total lockdown or partial easing of the lockdown to enable them procure food for their HH members. Moreover, it was difficult for them to look for short term jobs such as the daily wage jobs because these kind of jobs remained suspended during the partial lifting of COVID-19 lockdown [45]. Besides, HH heads who were tenants might have found it difficult to share their little or saved incomes between paying rent and buying food for HH members, which might have led to the significant association between poor food consumption status and HH heads who were tenants.

### Study strengths and limitations

Vaccines against COVID-19 disease have been released in several countries including Uganda. However, subsequent waves of COVID-19 outbreaks are expected in future because new variants are likely to evolve in the human population [58]. One strength of this present study is that it provides a baseline information about vulnerable HHs that may need social protection interventions such as food assistance to improve HH food consumption status amidst the ongoing partial COVID-19 lockdown restrictions and future COVID-19 total lockdowns in case subsequent waves of COVID-19 disease outbreaks emerge in Uganda [59]. Some limitations are also inherent in this present study. A reporting bias of food consumption is likely in this study. For example, HH heads might have over reported not eating any food sometimes in the previous week thinking that if they do so, they would get some form of food assistance during the COVID-19 lockdown, hence leading a higher prevalence of poor food consumption status.

Furthermore, this study has a potential of recall bias during data collection. For example, respondents might have forgotten to mention all the foods they consumed in the previous 7 days, leading to over estimation of poor food consumption status. It is worth noting that Uganda food aid recipients, mainly HHs headed by women were worried about on how they would cook food aid items such as common bean [36], which have a long cooking time [60]. Therefore, it is likely that some HHs that received food aid items did not consume it because they lacked fuel for cooking. However, this study did not rule out fuel availability for cooking received food items as an alternative explanation for the poor food consumption status. Furthermore, snowball sampling is prone to selection bias because initial subjects have a tendency of nominating participants they know well [61], for example their friends. Because of this, it is likely that the participants share the same traits and characteristics, thus, it is possible that the sample that we obtained may not have been representative of the entire population. Nevertheless, snowball sampling was necessary because it uses a chain referral process that allowed us reach participants that were difficult to sample amidst the COVID-19 restrictions including not allowing physical home visits to conduct face-face interviews.

## Conclusion

Food prices for staple foods, common bean and maize significantly increased during enforcing COVID-19 lockdown restrictions compared to before enforcing total lockdown restrictions in Nansana municipality. Food group consumption scores for staple legumes and cereals among slum HHs were significantly lower during partial lifting of COVID-19 lockdown compared to the period of COVID-19 total lockdown restrictions. In contrast, the FCS for non-slum HHs reduced during partial lifting compared to total COVID-19 lockdown. The prevalence of poor food consumption status increased among slum HHs during partial lifting of the COVID-19 lockdown restrictions compared to when the total lockdown was enforced. Slum HHs that received food aid items were less likely to face poor food consumption status than the other slum HHs that did not receive food aid. Households whose heads were females, daily wage earners, unemployed, and tenants were likely to face poor food consumption status either in slum or non-slum HHs. To reduce vulnerability to poor food consumption status, it was necessary to continue with food assistance in slum HHs during partial lifting of COVID-19 lockdown restrictions. Food assistance was necessary for HHs whose heads were females, daily wage earners, unemployed and tenants for either slum or non-slum HHs.

## Supplementary Information

**Additional file 1.** Fig. 1: A family receives food aid in Wabigalo-Namuwongo in urban Kampala on April 22 (30)

## Data Availability

The datasets used and/or analysed during the current study are available from the corresponding author on reasonable request.

## Abbreviations

AOR: Adjusted odds ratio
ANOVA: Analysis of variance
CI: Confidence interval
COR: Crude odds ratio
COVID-19: Coronavirus Disease 2019
FCS: Food Consumption Scores
HH: Household
HHs: Households
Ug Shs: Uganda shillings
