# Supplementary figures and images for: Food price volatility and socio-economic inequalities in poor food consumption status during coronavirus disease-2019 lockdown among slum and non-slum households in urban Nansana municipality, Uganda
Source: Nutr J. 2023 Jan 11;22:4. doi: 10.1186/s12937-023-00836-x (PMC9832412; doi:10.1186/s12937-023-00836-x)

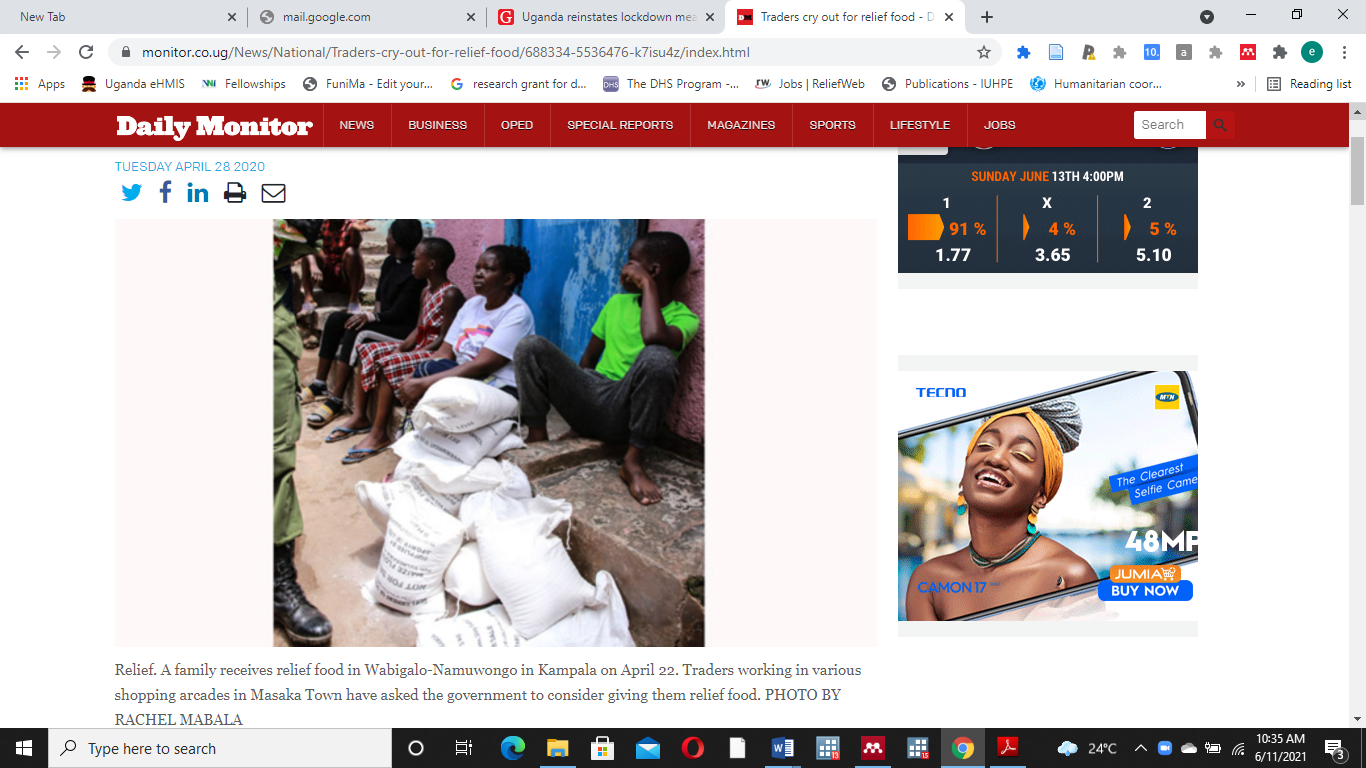


Figure 1: A family receives food aid in Wabigalo-Namuwongo in urban Kampala on April 22 (30).

Supplement: Supplementary file 1 — Additional file 1. Fig. 1: A family receives food aid in Wabigalo-Namuwongo in urban Kampala on April 22 (30) [file 12937_2023_836_MOESM1_ESM.docx]
